# STAT3 is constitutively activated in chronic active Epstein-Barr virus infection and can be a therapeutic target

**DOI:** 10.18632/oncotarget.25780

**Published:** 2018-07-24

**Authors:** Erika Onozawa, Haruna Shibayama, Honami Takada, Ken-Ichi Imadome, Sho Aoki, Mayumi Yoshimori, Norio Shimizu, Shigeyoshi Fujiwara, Takatoshi Koyama, Osamu Miura, Ayako Arai

**Affiliations:** ^1^ Department of Hematology, Graduate School of Medical and Dental Sciences, Tokyo Medical and Dental University (TMDU), Bunkyo-ku, Tokyo, Japan; ^2^ Department of Laboratory Molecular Genetics of Hematology, Graduate School of Medical and Dental Sciences, Tokyo Medical and Dental University (TMDU), Bunkyo-ku, Tokyo, Japan; ^3^ Department of Advanced Medicine for Infections, National Center for Child Health and Development (NCCHD), Setagaya-ku, Tokyo, Japan; ^4^ Center of Stem cell and Regenerative Medicine, Institute of Research, Tokyo Medical and Dental University (TMDU), Bunkyo-ku, Tokyo, Japan; ^5^ Department of Allergy and Clinical Immunology, National Center for Child Health and Development (NCCHD), Setagaya-ku, Tokyo, Japan; ^6^ Division of Hematology and Rheumatology, Department of Medicine, Nihon University School of Medicine, Itabashi-ku, Tokyo, Japan

**Keywords:** chronic active Epstein-Barr virus infection, STAT3, T/NK-cell lymphoproliferative disorder, cytokines, ruxolitinib

## Abstract

Chronic active Epstein-Barr virus infection (CAEBV) is a lymphoproliferative disorder characterized by the clonal proliferation of EBV-infected T or NK cells and is related to severe systemic inflammation. This study aims to investigate STAT3 to elucidate the mechanism underlying the CAEBV development. We determined that STAT3 was constitutively activated in EBV-positive T- or NK-cell lines. We also determined that STAT3 was activated in the peripheral blood mononuclear cells (PBMCs) containing EBV-infected clonally proliferating T or NK cells in six of seven patients with CAEBV. We conducted direct sequencing of the *STAT3* Src homology 2 (SH2) domain, which has previously been reported to be mutated in T- or NK-cell neoplasms. No mutation was detected in the *STAT3* SH2 domain in patients with CAEBV. Next, we investigated the effects of ruxolitinib, an inhibitor of both JAK1 and JAK2, which phosphorylates and activates STAT3. Ruxolitinib suppressed the phosphorylation of STAT3 in EBV-positive T- or NK-cell lines. Ruxolitinib also decreased the viable cell number of EBV-positive T- or NK-cell lines and PBMCs from patients with CAEBV. Furthermore, ruxolitinib suppressed the production of inflammatory cytokines in the cell lines and CAEBV patient-derived cells. In conclusion, constitutively activated STAT3, which promotes survival and cytokine production, could be a therapeutic target for CAEBV.

## INTRODUCTION

Chronic active Epstein-Barr virus infection (CAEBV) was first reported as sustained infectious mononucleosis accompanied by fever, lymphadenopathy, and liver dysfunction in patients without known immunodeficiency [1]. Later, it was reported that EBV-infected, clonally proliferating T or NK cells had been detected in patients with the disorder [2–4]. Furthermore, aggressive T- or NK-cell lymphoma can develop during the clinical course of CAEBV [5]. Hence, in the WHO classification, revised in 2017, CAEBV was classified into EBV-positive T- or NK-cell lymphoproliferative disorder [6].

The two characteristics of CAEBV are a T- or NK-cell lymphoproliferative disorder and an inflammatory disorder [7, 8]. Although EBV-infected T or NK cells in CAEBV clonally proliferate, they usually do not form solid tumors [8, 9]. Instead, they can be detected in the peripheral blood and can infiltrate into organs, leading to their dysfunction. Furthermore, the transformation of these cells can lead to chemotherapy-resistant T- or NK-cell lymphoma or leukemia [5, 8, 10]. The other aspect of CAEBV is an inflammatory disorder. The main symptoms of CAEBV are equivalent to those of chronic inflammation, such as persistent fever, lymphadenopathy, liver dysfunction with hepatosplenomegaly, vasculitis, neuritis, and dermatitis [5]. This chronic inflammation eventually could develop into the life-threatening disorder hemophagocytic lymphohistiocytosis (HLH).

Unfortunately, the mechanism underlying the CAEBV development remains unclear. At present, no effective agent exists for CAEBV, and the only curative treatment strategy has been allogeneic hematopoietic stem cell transplantation (allo-HSCT) [9, 11]. However, only a limited number of patients are suitable candidates for allo-HSCT. Furthermore, the outcomes of allo-HSCT for patients with an active disease, accompanied by fever, liver dysfunction, vasculitis, and uveitis, were significantly poorer than the outcomes for patients with an inactive disease [5, 9]. Hence, in order to establish effective medical treatments, it is imperative to determine the underlying mechanism of clonal proliferation of EBV-infected cells and sustenance of systemic inflammation in patients with CAEBV.

STAT3 is a transactivation factor that mediates proliferation and anti-apoptotic intracellular signaling. Constitutively activated STAT3 has been observed in numerous primary tumor cells as well as in tumor-derived cell lines obtained from patients, including those with lymphoid malignancies [12, 13]. STAT3 is constitutively activated in EBV-positive neoplasms, post-transplant lymphoproliferative diseases (LPDs) [14], EBV-positive diffuse large B-cell lymphoma (DLBCL) [15], nasopharyngeal cancer (NPC) cells [16], and extranodal NK/T-cell lymphoma (ENKL) [17]. In addition, Küçük et al. reported the presence of activating mutations in the Src homology 2 (SH2) domain of *STAT3* in EBV-positive γδ T- or NK-cell lines and in ENKL patient cells [18]. Interestingly, they also reported that a JAK1/2-specific inhibitor, AZD1480, inhibited the STAT3 activation as well as the proliferation of EBV-infected T- or NK-cell lines. As CAEBV is characterized by EBV-positive T- or NK-cells, we hypothesized that STAT3 was also constitutively activated in CAEBV. In addition, STAT3 induces inflammation by promoting the production of inflammatory cytokines, such as IFN-γ and TNF-ɑ, among others and by mediating the molecular signaling from their receptors [19]. This study aims to investigate STAT3 activation and its role in CAEBV using both cell lines and cells obtained from patients with CAEBV.

## RESULTS

### STAT3 is constitutively activated in EBV-positive T- or NK-cell lines

**Figure 1:**
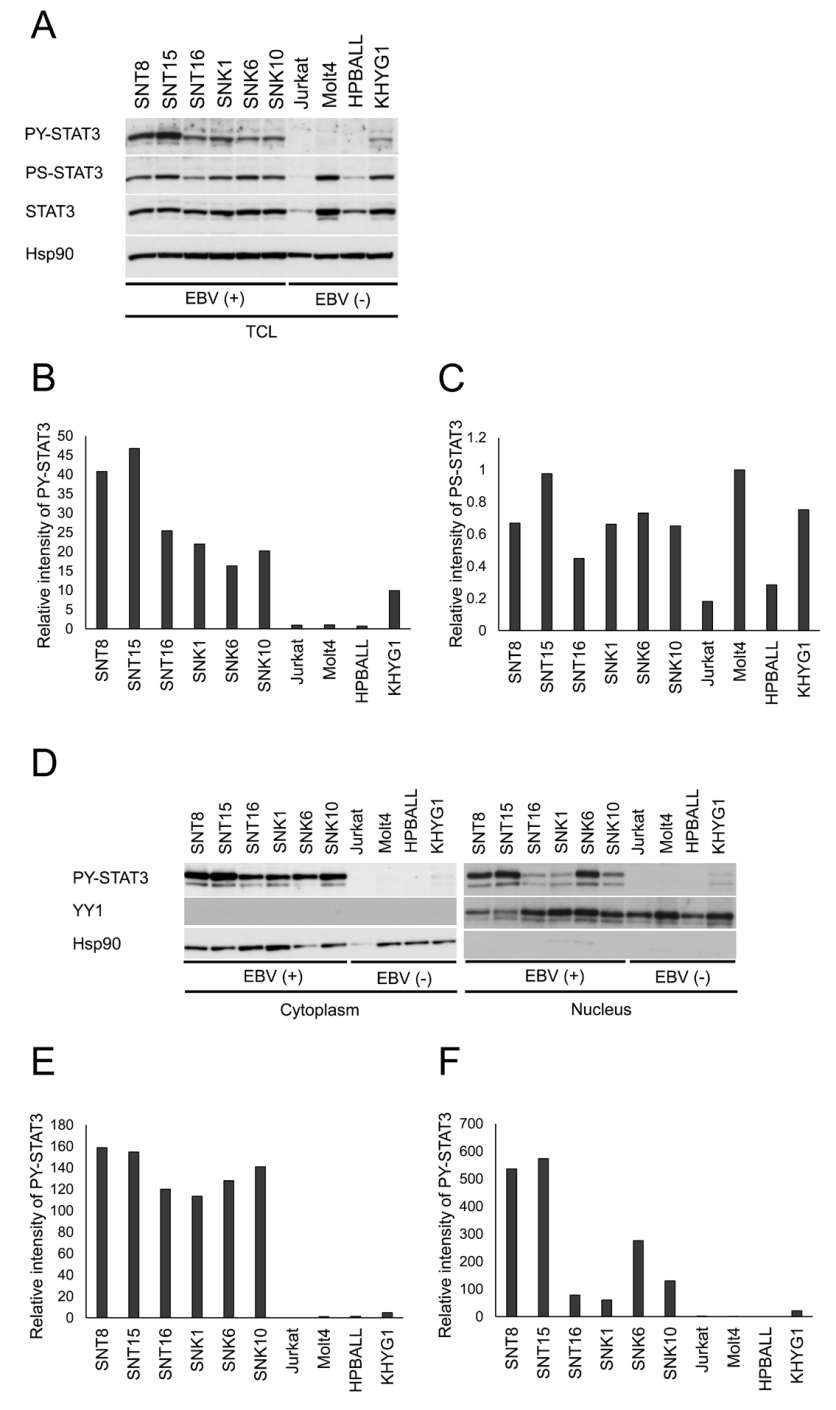
STAT3 is constitutively activated in EBV-positive T- or NK-cell lines **(A)** Western blotting for the phosphorylation of cell lines. Total cell lysates (TCL) were prepared, resolved by SDS-PAGE, and immunoblotted with antibodies, as indicated. STAT3 is constitutively phosphorylated in EBV-positive T- or NK-cell (EBV-T/NK-cell) lines but not in EBV-negative T- or NK-cell lines. Tyrosine-phosphorylated STAT3 (PY-STAT3) is detected in EBV-T/NK cell lines. Serine-phosphorylated STAT3 (PS-STAT3) is detected in all cell lines. EBV-negative cell lines do not exhibit or demonstrate a little phosphorylation of tyrosine. **(B** and **C)** the relative intensities of PY-STAT3 (B) and PS-STAT3 (C) bands of (A) were determined as ratio to total STAT3 by densitometry. MOLT4 was determined as a control. **(D)** Western blotting for STAT3 localization in EBV-T/NK-cell lines. Tyrosine-PY-STAT3 is localized in the nucleus in EBV-T/NK-cell lines but not in EBV-negative T- or NK-cell lines. Hsp90 and YY1 are proteins that were localized to the cytoplasm and nucleus, respectively. **(E** and **F)** the relative intensities of PY-STAT3 bands (D) of cytoplasm (E) and nucleus (F). The intensites were determined as ratio to Hsp90 (E) and YY1 (F), respectively by densitometry. MOLT4 was determined as a control.

We investigated the STAT3 activation in EBV-positive T- or NK-cell (EBV-T/NK-cell) lines established from patients with EBV-positive T- or NK-cell lymphoid neoplasm. For the activation of STAT3, the phosphorylation of both tyrosine-705 and serine-727 is indispensable. At first, we conducted an immunoblotting assay to determine the phosphorylation of STAT3 (Figure 1A). Figures 1B and 1C show the relative intensity of the bands by the densitometry analysis. The serine-727 phosphorylation of STAT3 was detected in all cell lines under the maintenance condition (Figures 1A and 1C). However, the phosphorylation of tyrosine-705 was detected in EBV-positive T- or NK-cells, not in Jurkat, MOLT4, and HPB-ALL cells, which are EBV-negative T-cell lines (Figures 1A and 1B). In KHYG1 cells, an EBV-negative NK-cell line, a little phosphorylation of tyrosine-705 of STAT3 was detected (Figures 1A and 1B). In addition, we investigated the localization of STAT3 in these cells, as activated STAT3 is phosphorylated and localized in the nucleus. Figure 1D shows that STAT3 was phosphorylated and detected in the cytoplasmic and nuclear fraction in EBV-T/NK-cell lines by western blotting. Figures 1E and 1F show the densitometry analysis. EBV-negative cell lines did not exhibit tyrosine-phosphorylated STAT3 in the nucleus under these conditions (Figures 1D, 1E and 1F).

### STAT3 is constitutively activated in EBV-positive T- or NK-cells from patients with CAEBV

**Figure 2:**
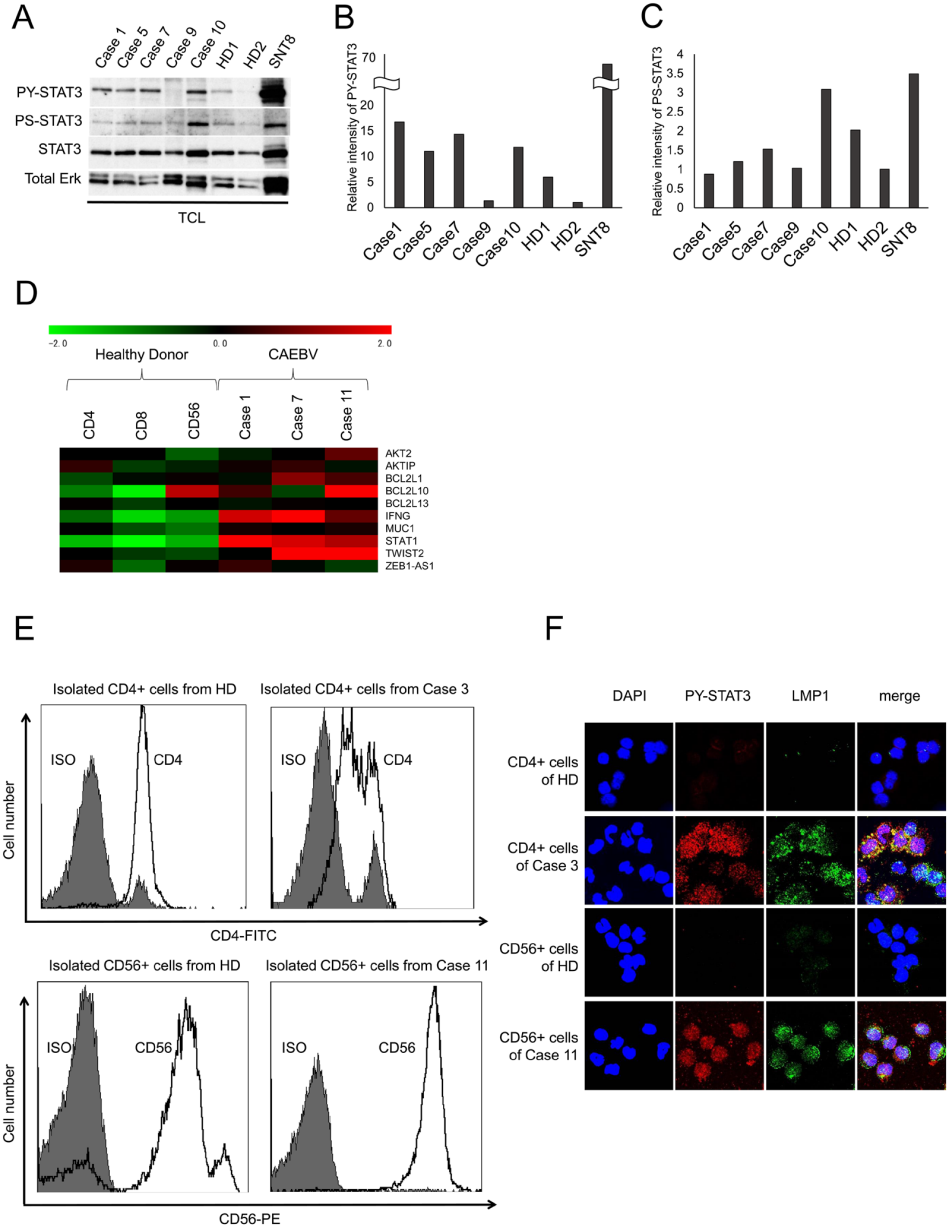
STAT3 is constitutively activated in patient-derived EBV-infected T or NK cells **(A)** Western blotting for the phosphorylation of the PBMCs obtained from patients with CAEBV. Total cell lysates (TCL) were prepared, resolved by SDS-PAGE, and immunoblotted with antibodies, as indicated. STAT3 is constitutively phosphorylated in the PBMCs from patients with CAEBV, except for Case 9. The phosphorylation was relatively weak or not detected in cells from healthy donors (HD). SNT8 is a positive control. **(B** and **C)**; the relative intensities of tyrosine-phosphorylated STAT3 (PY-STAT3) (B) and serine-phosphorylated STAT3 (PS-STAT3) (C) bands of (A) were determined as ratio to total STAT3 by densitometry. HD 2 was determined as a control. **(D)** the expression of STAT3 responsible genes in the T- or NK-cell fractions of the PBMCs containing EBV-infected cells from CAEBV patients. The EBV-infected cell fractions were CD4-, CD8-, and CD56-positive cells from Cases 1, 7, and 11, respectively. The same cell fractions from HD were examined as EBV-negative controls. A heat map shows the expression levels from genes induced by STAT3 in the patient-derived PBMCs fractions containing EBV-infected cells and control samples. **(E** and **F)** STAT3 activation in EBV-positive and CD4- or CD56-positive cells from patients with CAEBV. CD4- and CD56-positive cells were isolated using anti-CD4 and anti-CD56 antibody-conjugated magnetic beads from the peripheral blood of Cases 3, 11, respectively. The same cell fractions from HD were examined as EBV-negative controls. (E) surface CD4 and CD56 expression is confirmed by flow cytometry using antibodies to CD4 and CD56 (open histogram). The gray, shaded histograms are isotype-matched control immunoglobulin. Top, Case 3; bottom, Case 11. (F) the tyrosine phosphorylation of STAT3 in isolated cells is demonstrated by immunofluorescence staining. Nuclear as well as cytoplasmic localization of STAT3 is detected in patients but not in HDs.

We validated the results mentioned above in patient-derived cells. In CAEBV, EBV-positive cells are detected in the peripheral blood. In this study, 14 patients with CAEBV (aged 18-64 years; five males, nine females; CD4 type: *n* = 4; CD8 type: *n* = 4; CD56 type: *n* = 3; CD4 and CD56 double infection: *n* = 2; and CD4 and CD8 double infection: *n* = 1) were investigated. Table 1 presents the clinical findings, phenotype, and EBV DNA load of infected cells. The clonal proliferation of infected cells was detected in the peripheral blood mononuclear cells (PBMCs) of all patients. The EBV DNA load of the patient-derived PMBCs was 1.7×10^3^-2.6×10^5^ (mean: 9.2 × 10^4^) copies/μg DNA. We studied the STAT3 activation in the PBMCs obtained from five patients; the cells were isolated from the peripheral blood, immediately lysed, and used for western blotting (Figure 2A). Figures 2B and 2C show the relative intensity of the bands by the densitometry analysis. The lysate of SNT8 cells was a positive control. The phosphorylation of serine-727 was detected in the samples from patients as well as those from healthy donors. On the other hand, tyrosine-705 of STAT3 was significantly phosphorylated in four of five patient cells. The non-detection of the tyrosine phosphorylation in Case 9 could present the negative or weak activation of STAT3, or could be attributed to the low rate of EBV-positive cells in the PBMCs (Table 1), because STAT3 was constitutively expressed in PBMCs. Taken together, the phosphorylation of both tyrosine-705 and serine-727 was detected in patients’ samples. In addition, we investigated the expression of STAT3-inducible genes by the microarray analysis. The fractions containing EBV-infected cells were isolated from the PBMCs of patients, Cases 1, 7, and 11, whose EBV-positive cells were CD4-, CD8-, and CD56-positive cells, respectively. For the isolation, we used antibody-conjugated magnetic beads against surface markers on infected cells. In addition, the RNA was extracted from cells and used for the microarray analysis. Figure 2D shows that the expression of STAT3-inducible genes, including *IFN-γ*, was generally enhanced in the patient-derived T- or NK-cell fractions that included EBV-positive cells. Furthermore, we examined the activation and localization of STAT3 in EBV-positive T- or NK-cells from the patients. In Cases 3 and 11, EBV-infected cells were CD4- and CD56-positive, respectively (Table 1). We isolated CD4- or CD56-positive cells from the PBMCs of patients and healthy donors using antibody-conjugated magnetic beads. Figure 2E shows the flow cytometry for the isolated cells, confirming that a majority of the isolated cells were positive for CD4 or CD56. Figure 2F shows the localization of tyrosine-phosphorylated STAT3 (PY-STAT3) in isolated CD4- or CD56-positive cells. PY-STAT3 was detected not only in the cytoplasm but also in the nucleus of patient-derived CD4- or CD56-positive cells, whereas it was not detected in CD4- or CD56-positive cells from healthy donors. EBV infection was determined by the LMP1 expression in patient-derived CD4- or CD56-positive cells. These results indicated that STAT3 was constitutively activated in PBMCs fractions containing EBV-infected T or NK cells in CAEBV. In Case 3 and 11, STAT3 was constitutively activated in EBV-infected T or NK cells. At present, however, it is not clear whether this activation is specific to EBV-infected cells, because we could not examine infected and un-infected cells separately.

**Table 1:** The characteristics of patients

Case	Gender	Age	EBV-Infected cells	Clinical findings	Clonality of the EBV-infected cells	EBV DNA load (copies/μg DNA) of the whole blood	EBV DNA load (copies/μg DNA) of the EBV-infected cells fraction in the PBMCs	The rate of the EBV-infected cell fractions in the PBMCs
1	F	25	CD4	Fever, liver dysfunction, lymphadenopathy	Monoclonal	7.0 × 10^4^	2.2 × 10^5^ (CD4)	71.2%
2	M	45	CD4	Fever, sMBA	Monoclonal	1.7 × 10^3^	1.7 × 10^4^ (CD4)	NE
3	M	34	CD4	Fever	Monoclonal	5.7 × 10^4^	4.9 × 10^5^ (CD4)	54.3%
4	M	20	CD4	Fever, liver dysfunction, lymphadenopathy	Monoclonal	2.0 × 10^5^	1.5 × 10^5^ (CD4)	60.5%
5	F	38	CD8	Fever, liver dysfunction, lymphadenopathy	Monoclonal	1.4 × 10^5^	3.9 × 10^5^ (CD8)	76.5%
6	F	21	CD8	Fever, liver dysfunction	Monoclonal	4.7 × 10^4^	1.7 × 10^4^ (CD8)	50.0%
7	F	64	CD8	Fever, liver dysfunction, lymphadenopathy	Monoclonal	2.6 × 10^5^	1.2 × 10^6^ (CD8)	51.0%
8	M	28	CD8	Fever, liver dysfunction, lymphadenopathy, neuritis	Monoclonal	1.9 × 10^3^	4.1 × 10^5^ (CD8)	NE
9	F	18	CD56	sMBA	Monoclonal	5.2 × 10^4^	1.6 × 10^6^ (CD56)	3.8%
10	F	48	CD56	Fever, sMBA, HLH	Monoclonal	8.6 × 10^4^	1.6 × 10^5^ (CD56)	10.0%
11	F	23	CD56	sMBA	Monoclonal	5.1 × 10^4^	4.6 × 10^5^ (CD56)	17.9%
12	M	37	CD4, CD56	Fever, lymphadenopathy	Monoclonal	1.9 × 10^5^	1.9 × 10^5^ (CD4),1.1 × 10^6^ (CD56)	30.0% (CD4), 0.2% (CD56)
13	F	18	CD4, CD56	Fever, polyneuropathy,	Monoclonal	6.0 × 10^4^	1.7 × 10^5^ (CD4), 7.1 × 10^6^ CD56)	35.2% (CD4), 2.3% (CD56)
14	F	22	CD4, CD8	Fever, HV	Monoclonal	7.2 × 10^4^	1.8 × 10^4^ (CD4), 1.4 × 10^5^ (CD8)	41.2% (CD4), 51.7% (CD8)

F, female; M, male; NE, not examined

EBV; Epstein-Barr virus, PBMCs; peripheral blood mononuclear cells, F; female, M; male, sMBA; severe mosquito bite allergy, NE; not examined, HLH; Hemophagocytic lymphohistiocytosis, HV; hydroa vacciniforme-like eruption

### Mutation analysis of the STAT3 SH2 domain in EBV-positive T- or NK-cells

The SH2 domain of *STAT3* frequently exhibits gain-of-function mutations in large granular lymphocytic leukemia. Mutations in this domain have also been identified in γδ T cells from lymphoid neoplasms including ENKL [18, 20]. Hence, we investigated the mutation status of the SH2 domain in EBV-positive T- or NK-cells from patients with CAEBV. Three of six EBV-positive T- or NK-cell lines: SNT8; SNT15; and SNK6, presented a mutation in the SH2 domain of *STAT3* (D661Y in SNT8 and SNK6, and Y640F in SNT15; Table 2; Supplementary Figure 1). These mutations were identical to those previously reported [18]. However, no mutation was detected in the PBMCs analyzed, which included EBV-infected and clonally proliferating T or NK cells from 14 patients with CAEBV.

**Table 2:** Sequence analysis of the SH2 domain of STAT3 of EBV-positive T- or NK-cell lines

Cell lines	Exon 19	Exon 20	Exon 21
SNT8	WT	WT	D661Y
SNT15	WT	WT	Y640F
SNT16	WT	WT	WT
SNK1	WT	WT	WT
SNK6	WT	WT	D661Y
SNK10	WT	WT	WT

### The effects of JAK1/2 inhibitors on the phosphorylation of STAT3 in EBV-positive T- or NK-cell lines

**Figure 3:**
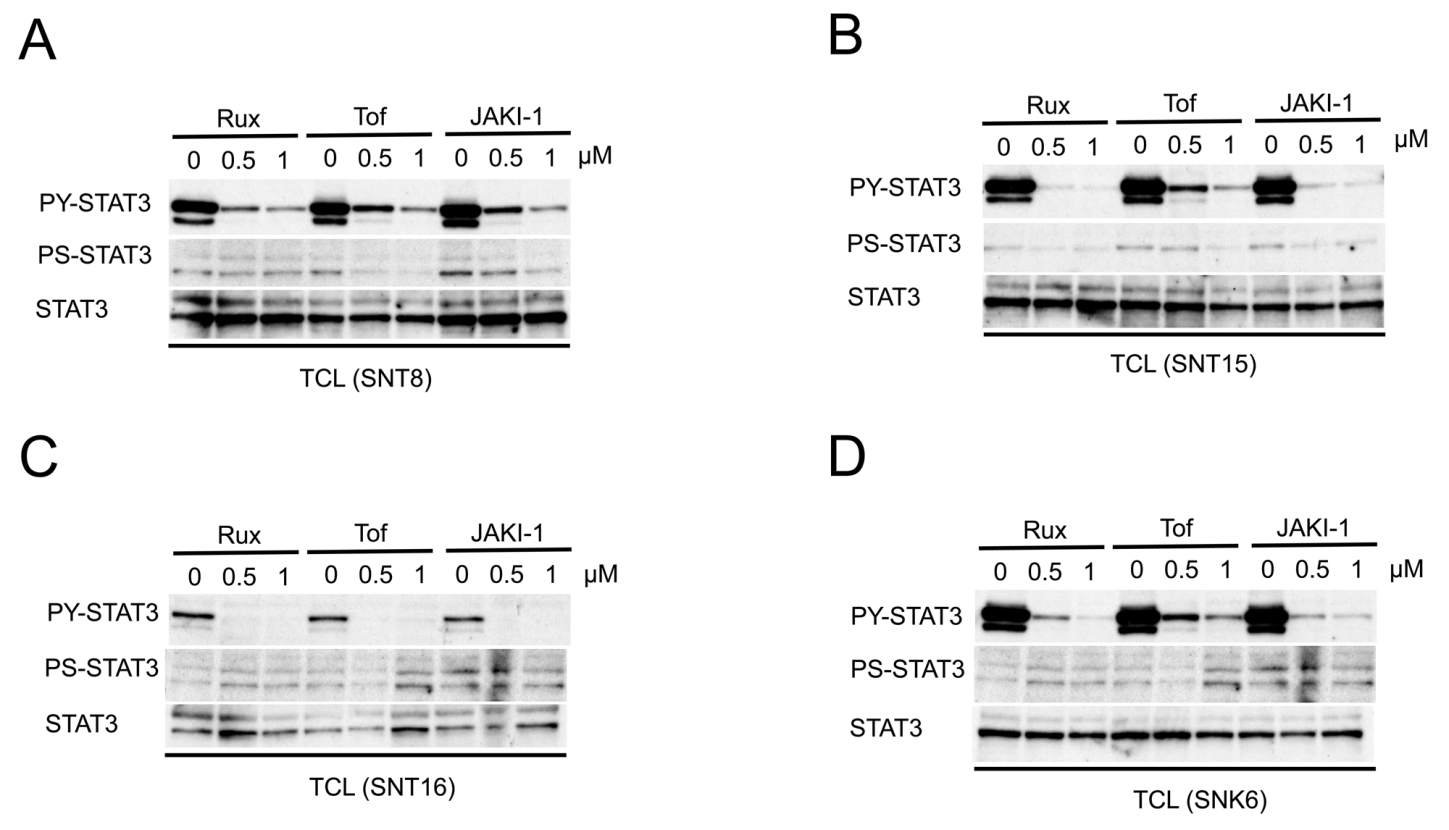
JAK inhibitors suppress the constitutive phosphorylation of STAT3 in EBV-positive T- or NK-cell lines **(A-D)** Western blotting for the phosphorylation of cell lines treated with JAK inhibitors. Total cell lysates (TCL) were prepared, resolved by SDS-PAGE, and immunoblotted with antibodies, as indicated. The phosphorylation of tyrosine-705-STAT3 in cell lines was suppressed by JAK inhibitors in a dose-dependent manner: SNT8 (A); SNT15 (B); SNT16 (C); and SNK6 (D). The phosphorylation of serine-727 was not clearly suppressed. Rux, ruxolitinib; Tof, tofacitinib; JAKI1, Jak inhibitor 1.

The results described above suggest that the constitutive activation of STAT3 might be induced by an upstream intracellular signaling molecule. JAKs are kinases that phosphorylate and activate STAT3. Hence, we investigated the effects of JAK inhibitors on the phosphorylation of STAT3 in EBV-positive T- or NK-cell lines. Interestingly, the tyrosine-705 phosphorylation of STAT3 was inhibited by ruxolitinib (a JAK1/2 inhibitor), tofacitinib (a JAK3 inhibitor), and JAK inhibitor 1 (a JAK1/2/3 inhibitor) in a dose-dependent manner even in cell lines harboring the mutation (Figure 3). The phosphorylation of serine-727 of STAT3 was not clearly suppressed after the treatment. Dimethyl sulfoxide (DMSO), a solvent for inhibitors, did not exhibit significant effects on the phosphorylation (Supplementary Figure 2). Thus, even in cells with the SH2 domain mutation, the constitutive activation of STAT3 could be inhibited in EBV-positive T- or NK-cell lines by the inhibition of JAKs.

### The biological effects of ruxolitinib on EBV-positive T- or NK-cell lines and CAEBV patient-derived cells

**Figure 4:**
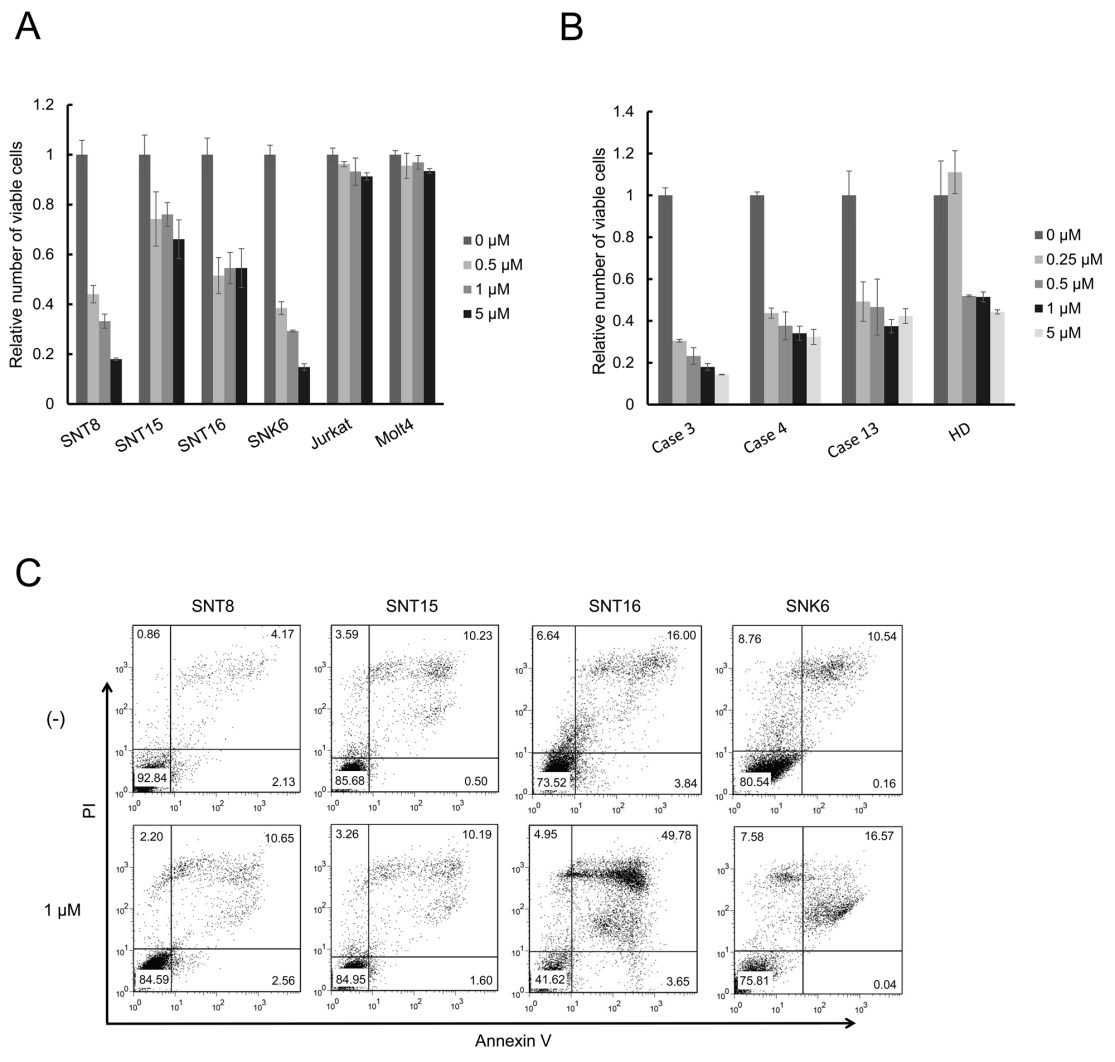
Ruxolitinib suppresses viable cell number of EBV-infected T or NK cells **(A)** EBV-infected T- or NK-cell lines were treated with ruxolitinib, as indicated, for 48 h, and the number of viable cells was estimated by an XTT assay and expressed in arbitrary units. The data are shown as mean±standard deviations (SD) of three independent experiments. **(B)** the PBMCs from 3 patients with CAEBV and 1 healthy donor were treated with ruxolitinib, as indicated, in the presence of IL-2 for 72 h, and the viable cell number was estimated by an XTT assay and expressed in arbitrary units. The data are presented as mean±SD of three independent experiments. **(C)** EBV-infected T- or NK-cell lines were treated with ruxolitinib for 48 h, as indicated, and then analyzed. Cells were stained with Annexin V and PI and subsequently analyzed by flow cytometry.

**Figure 5:**
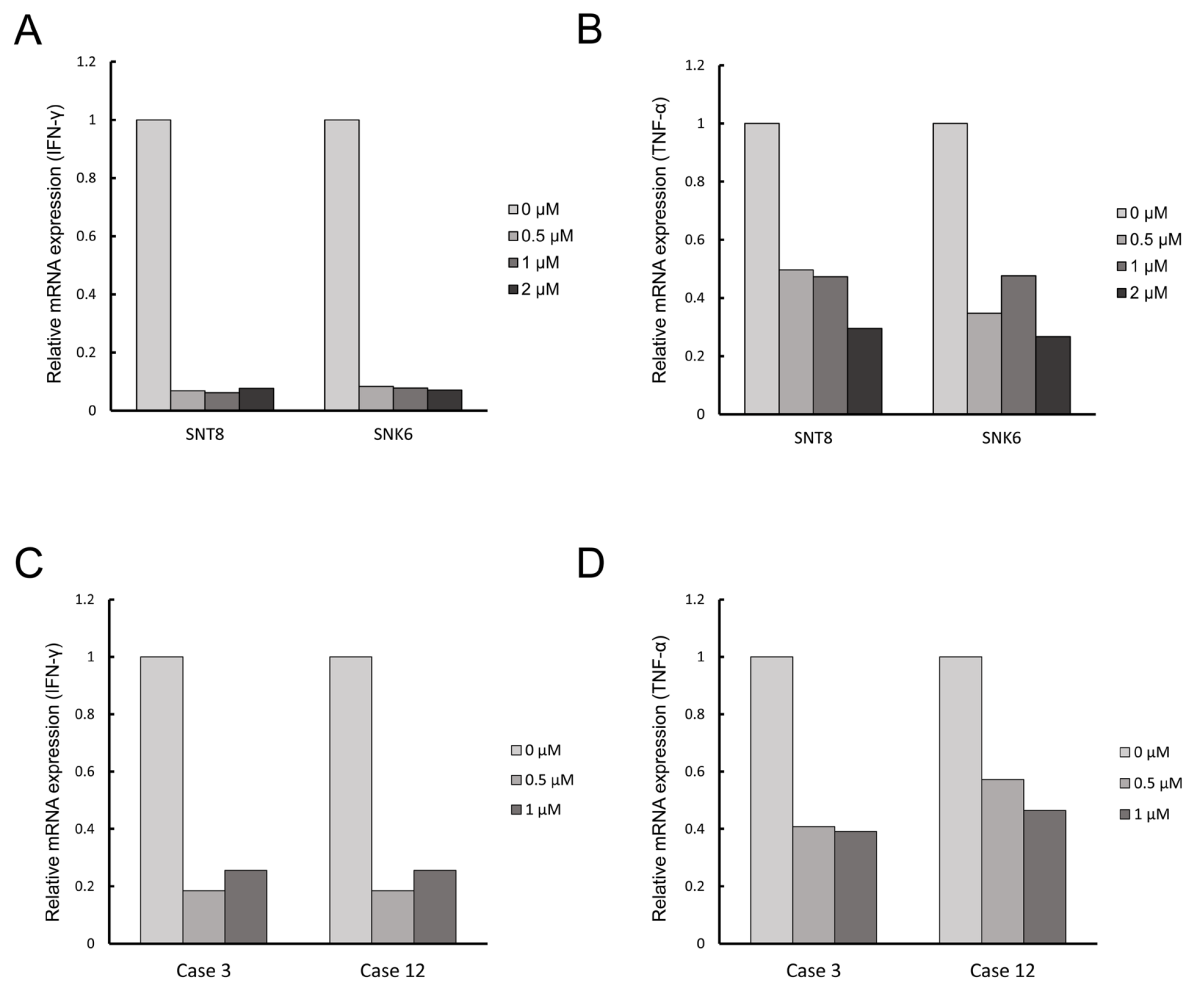
Ruxolitinib suppresses the mRNA expression of inflammatory cytokines in EBV-infected T- or NK-cells **(A** and **B)** EBV-infected T- or NK-cell lines were treated with ruxolitinib, as indicated, for 24 h. The RNA was extracted and subjected to quantitative PCR assays. (A) IFN-γ; (B) TNF-ɑ; **(C** and **D)**, the PBMCs from patients with CAEBV were treated with ruxolitinib, as indicated, for 24 h. The RNA was extracted and used for quantitative PCR assays. (C) IFN-γ; (D) TNF-ɑ.

From the three inhibitors, we selected ruxolitinib, the effective concentration of which was identical to that of its clinical use. We investigated the effects of ruxolitinib on the proliferation and survival of EBV-positive T- or NK-cells. The XTT assay demonstrated that ruxolitinib suppressed the viable cell number of EBV-positive T- or NK-cell lines in a dose-dependent manner (Figure 4A). In addition, ruxolitinib suppressed a viable cell number of CAEBV patient-derived PBMCs in a dose-dependent manner (Figure 4B). Ruxolitinib also suppressed the number of healthy donor-derived PBMCs. However, under the concentration of 0.25 μM, which can be actually achieved in patients, the number of patient-derived PBMCs was decreased whereas that of healthy donor-derived PBMCs was not. As shown in Figure 4C, ruxolitinib induced cellular apoptosis in SNT16, although it was not remarkable in SNT8, SNT15, and SNK6 cells. Finally, we investigated the effects on cytokine production. Figures 5A and 5B show that ruxolitinib suppressed the mRNA production of inflammatory cytokines, IFN-γ and TNF-ɑ in EBV-positive T- or NK-cell lines. Furthermore, ruxolitinib suppressed the mRNA production of inflammatory cytokines IFN-γ (Figure 5C) and TNF-ɑ (Figure 5D) in the CAEBV patient-derived PBMCs. The mRNA of interleukin-6 (IL-6) was not detected in these cells (data not shown). The viability of each cells was approximately 80%, and 60-80%, in the cell lines and patients’ cells, respectively (Supplementary Figure 3).

## DISCUSSION

Our results indicated that STAT3 was constitutively activated in EBV-positive T- or NK-cells from patients with CAEBV. This study did not detect activating mutations of STAT3 in these cells. Furthermore, the tyrosine phosphorylation of STAT3, as well as the survival, was suppressed by ruxolitinib even in cell lines harboring the activating mutation of STAT3. Although mutations detected in the SH2 domain upregulated the dimerization of STAT3 [18], our results indicated that the activation of STAT3 depended on the tyrosine phosphorylation.

The underlying mechanism for the phosphorylation of STAT3 in CAEBV could be attributed to several factors. First, the activation of JAK kinases which directly phosphorylates and activates STAT3, should be examined. Previously, gain-of-function mutations have been reported in JAK1 or JAK3 in some neoplasms, leukemia, breast cancer, lung cancer, hepatocellular carcinoma [21], and NK-lymphoma cells [22–24]. Interestingly, an association between the JAK1 expression and the STAT3 activation has been reported in T-cell lymphomas [25]. In addition, Src family kinases can activate STAT3. Reportedly, LMP1 activates Src kinases in EBV-infected B-cells [26], and this activity should be investigated in EBV-positive T- or NK-cells in CAEBV.

Several studies have suggested that STAT3 might be activated downstream of LMP1 through the activation of NF-κB. In EBV-infected B cells or epithelial cells, NF-κB is activated by C-terminal-activating region 1 (CTAR1) of LMP1 [27, 28]. In addition, we recently determined that NF-κB was constitutively activated through LMP1, promoting survival in EBV-positive T- or NK-cells obtained from patients with CAEBV [29]. Chen et al. reported that NF-κB activates STAT3 through IL-6 production in EBV-infected epithelial cells [30]. They mentioned that IL-6 associates with IL6R/gp130 expressed on cells and activates STAT3 through the JAK activation in cells by autocrine mechanisms. In addition, IL-6 is elevated in the serum of patients with CAEBV [19]. Although the mRNA of IL-6 was not detected in EBV-positive T- or NK-cell lines in this study, IL-6 can be produced by other inflammatory cells such as macrophages in CAEBV. Reportedly, activated NF-κB induces the expression of a receptor tyrosine kinase epidermal growth factor receptor (EGFR), leading to the STAT3 activation by the phosphorylation on Y-705 in NPC cells. According to the study by Nosbaum et al., EGFR can be inducibly expressed on T or NK cells [31]. Thus, EGFR could also mediate the STAT3 activation in EBV-positive T- or NK-cells. The expression and the activation of EGFR in CAEBV, and the effects of ruxolitinib on EGFR warrant further investigation. Overall, NF-κB might induce STAT3 activation through IL-6 or EGFR in EBV-infected T or NK cells. Furthermore, other molecular pathways can contribute to the activation of STAT3 in EBV-infected T or NK cells. Erk is reportedly activated downstream of LMP1 and induces the serine phosphorylation of STAT3 [32]. Our recent study determined that Erk was constitutively activated in EBV-infected T or NK cells (our unpublished data). Additional studies in this regard are ongoing in our laboratory.

In this study, we determined that ruxolitinib suppresses the phosphorylation of STAT3, decreasing the survival and cytokine production in EBV-positive cells of patients with CAEBV. However, ruxolitinib-induced apoptosis was not clear in SNT8, SNT15 and SNK6 cells (Figure 4C). Additional mechanisms, such as necrosis or other types of programmed cell death, might be induced by the treatment. It is indispensable to confirm the effects using PBMCs of patients with CAEBV.

The two features of CAEBV are a neoplastic disease and an inflammatory disease. Since the concentration of inflammatory cytokines in the serum of patients with CAEBV is closely related to the disease status [19], these results suggest that ruxolitinib is effective for CAEBV treatment by suppressing both neoplastic cells and inflammation. Interestingly, two studies recently reported that JAK1/2 inhibition was effective in treating a mouse model of HLH [33, 34]. HLH, a severe form of inflammation, occurs in 24% of patients with CAEBV and can be lethal [9]. Inhibition of the JAK/STAT pathway might inhibit the development of HLH and could be an attractive candidate for CAEBV treatment. Nonetheless, further extensive studies using clinical samples are needed to confirm the results and apply these findings clinically.

## MATERIALS AND METHODS

### Cells and reagents

EBV-T/NK-cell lines SNT8, SNT15, SNT16, SNK1, SNK6, and SNK10 were cultured in RPMI-1640 containing 10% fetal calf serum (10% FCS-RPMI) and 175 U/mL human IL-2 [35]. The EBV-negative T- and NK-cell lines Jurkat, MOLT4, and HPB-ALL were cultured in 10% FCS-RPMI, whereas the EBV-negative NK-cell line KHYG1 was cultured in 10% FCS-RPMI containing 175 U/mL human IL-2. IL-2 was purchased from R&D Systems (Abington, UK). Ruxolitinib and tofacitinib were purchased from LC Laboratories (Boston, MA). Jak inhibitor 1 was purchased from Merck Millipore (MA). DMSO was purchased from Wako Pure Chemical Industries (Osaka, Japan).

### Diagnosis of CAEBV

CAEBV was diagnosed on the basis of following criteria: (1) the presence of characteristic clinical findings such as persistent systemic inflammation, fever, liver dysfunction, lymphadenitis, vasculitis, uveitis, severe mosquito bite allergy (sMBA), and hydroa vacciniforme-like eruption (HV); (2) a high EBV load detected in the PBMCs by quantitative PCR (> 10^2.5^ copies/μg of EBV DNA); and (3) EBV infection in T or NK cells [5, 36]. The clonality of the infected cells was examined by Southern blotting.

### Detection of EBV-infected cells in patients with CAEBV

Infected cells were detected and isolated as described previously [37]. Briefly, the PBMCs obtained from patients were isolated by density gradient centrifugation using Separate-L (Muto Pure Chemical, Tokyo, Japan) and were sorted into CD19-, CD4-, CD8-, or CD56-positive fractions using antibody-conjugated magnetic beads (Miltenyi Biotec, Bergisch Gladbach, Germany; 130-050-301, 130-045-101, 130-045-201, 130-090-875). After that, the EBV DNA levels in each fraction were evaluated by real-time PCR using the TaqMan System (Applied Biosystems, Foster City, CA) [38].

### Detection of the clonality

The clonal proliferation of EBV-infected cells was detected by Southern blotting for EBV-terminal repeat. [39].

### Isolation of the EBV-infected cell fraction from patients with CAEBV

For assays, we isolated the fraction containing EBV-infected cells from the PBMCs using antibody-conjugated magnetic beads against a surface marker on infected cells (IMag Anti-Human CD4, 8, or 56 Particles-DM; BD Biosciences). Table 1 describes the marker used to isolate infected cells from each patient.

### Apoptosis detection assay

Apoptosis was detected using a TACS^®^ Annexin V-FITC apoptosis detection kit (Trevigen, Gaithersburg, MD), according to the manufacturer’s instructions.

### XTT assay

The XTT assay was performed according to the sodium 3V-(1-[phenylaminocarbonyl]-3,4-tetrazolium)-bis(4-methoxy-6-nitro)-benzene sulfonic acid hydrate (XTT) colorimetric method using the Cell Proliferation Kit II (Roche Molecular Biochemicals, Indianapolis, IN), according to the manufacturer’s instructions.

### Antibodies

For western blotting, we used the following antibodies: phospho-STAT3 (Tyr705, No.9145); phospho-STAT3 (Ser727, No.9134; Cell Signaling Technology, Danvers, MA); and Hsp90ɑ/β (F-8, No.SC13119; Santa Cruz Biotechnology, CA). For immunofluorescence staining, we used an antibody against STAT3 (H-190, No.SC7179; Santa Cruz Biotechnology).

### Cell lysis for western blotting

The assay was performed as described previously [40]. For total cell lysis, cells were lysed in SIP buffer (50 mM Tris-HCl [pH 7.5], 5 mM EDTA, 100 mM NaCl, 50 mM NaF, 1 mM Na_3_VO_4_, 40 mM β-glycerophosphate, and 1% Triton X-100) after washing with phosphate buffered saline (PBS). The resulting lysate was centrifuged at 15,000 rpm for 10 min, and the supernatant was collected and subjected to western blotting.

Furthermore, proteins were separated into cytoplasmic and nuclear fractions, as described later. After washing with PBS, cells were lysed in hypotonic buffer (50 mM Tris-HCl [pH 7.5], 5 mM EDTA, 10 mM NaCl, 1 mM NaF, 1 mM Na_3_VO_4_, and 0.05% NP-40). Lysates were centrifuged at 700×*g* for 2 min, and supernatants were collected as cytoplasmic protein fractions. Pellets were washed three times with hypotonic buffer and lysed in SIP buffer (50 mM Tris-HCl [pH 7.5], 5 mM EDTA, 100 mM NaCl, 50 mM NaF, 1 mM Na_3_VO_4_, 40 mM β-glycerophosphate, and 1% Triton X-100). Lysates were centrifuged at 15,000 rpm for 10 min, and supernatants were used as nuclear protein fractions. Proteins were quantified using the DC protein assay kit (Bio-Rad, Hercules, CA) and equivalent protein quantities were subjected to western blotting.

The relative intensities of bands were determined by densitometry using ImageJ software (https://imagej.nih.gov/ij/).

### Immunofluorescence staining

Cells were fixed on slides by immersion in 4% paraformaldehyde for 10 min, followed by three washes in PBS and were incubated with mouse monoclonal anti-LMP1 and rabbit polyclonal anti-phospho-STAT3 (Tyr705) antibodies at room temperature. Then, the slides were treated at room temperature with a Cy5-conjugated AffiniPure donkey anti-mouse antibody (No.715-175-151, Jackson ImmunoResearch Laboratories, Inc., West Grove, PA) to label the anti-LMP1 antibody and a PE-conjugated goat anti-rabbit antibody (No. 4030-09; Southern Biotech, Birmingham, AL) to label the anti-phospho-STAT3 antibody. The nuclei were counterstained with ProLong Gold and DAPI (Invitrogen, Carlsbad, CA).

### Microarray analysis

The total RNA was extracted from the EBV-infected cell fraction of the PBMCs obtained from patients with CAEBV using TRIzol Reagent (Thermo Fisher Scientific K.K, Waltham, MA) and purified using the SV Total RNA Isolation System (Promega, Madison, WI), according to the manufacturer’s instructions. We quantified RNA samples by an ND-1000 spectrophotometer (NanoDrop Technologies, Wilmington, DE) and confirmed the quality with the Experion System (Bio-Rad Laboratories). The cRNA was amplified, labeled using the Low Input Quick Amp Labeling Kit, and hybridized to a SurePrint G3 Human Gene Expression Microarray 8×60K v2 (Agilent Technology, Santa Clara, CA), according to the manufacturer’s instructions. All hybridized microarray slides were scanned by an Agilent scanner. Furthermore, relative hybridization intensities and background hybridization values were calculated using Agilent Feature Extraction Software (9.5.1.1; Agilent Technology).

We calculated raw signal intensities and flags for each probe from hybridization intensities (gProcessedSignal) and spot information (gIsSaturated), according to the manufacturer’s instructions (Agilent). In addition, raw signal intensities were normalized by a quantile algorithm using the “preprocessCore” library package in the Bioconductor software [41, 42].

### Flow cytometry

The PBMCs were isolated and analyzed by a FACS Calibur flow cytometer (Becton Dickinson and Company, Franklin Lakes, NJ) with FITC-conjugated mouse anti-human CD4 and PE-conjugated mouse anti-human CD56 antibodies (No.561842, No.555516; Becton Dickinson and Company).

### qRT-PCR

The RNA was extracted with ISOGEN II (Nippon Gene, Tokyo, Japan) from the cell lines and PBMCs, and cDNA was generated using Transcriptor Universal cDNA Master (Roche, Basel, Switzerland). The DNase treatment was performed using shrimp DNase recombinant (Affymetrix, Santa Clara, CA). In addition, we performed qRT-PCR analyses on a Light Cycler 480 (Roche) using TaqMan^®^ Gene Expression Assays (Applied Biosystems). The primers for interferon-γ and TNF-ɑ were Hs00989291_m1 and Hs01113624_g1, respectively.

### DNA sequencing

In this study, genomic DNA was extracted using the DNeasy Blood & Tissue Kit (QIAGEN, Hilden, Germany). PCR analyses of genomic DNA were performed in a 50 μL reaction volume containing 5× prime STAR GXL Buffer, a dNTP mixture, a forward primer (10 μM), a reverse primer (10 μM), and Prime STAR GXL DNA polymerase (TAKARA BIO, Shiga, Japan); the primers used in this study have been previously described [20]. The PCR product was run on an agarose gel, excised with a scalpel, and purified using the MinElute Gel Extraction Kit (QIAGEN, Hilden, Germany). Furthermore, sequencing was performed using Ion PGM (Life Technologies, CA).

### Ethics statement

The study was conducted in accordance with the principles of the Declaration of Helsinki and was approved by the ethical committee of the Tokyo Medical and Dental University (TMDU, Tokyo, Japan). We obtained written informed consent from all patients before enrollment in this study.

## SUPPLEMENTARY MATERIALS FIGURES


