# Supplementary material for: STAT3 is constitutively activated in chronic active Epstein-Barr virus infection and can be a therapeutic target
Source: Oncotarget. 2018 Jul 24;9(57):31077–89. doi: 10.18632/oncotarget.25780 (PMC6089567; doi:10.18632/oncotarget.25780)
Supplement: Supplementary file 1 [file oncotarget-09-31077-s001.pdf]

STAT3 is constitutively activated in chronic active Epstein-Barr virus infection and can be a therapeutic target

SUPPLEMENTARY MATERIALS

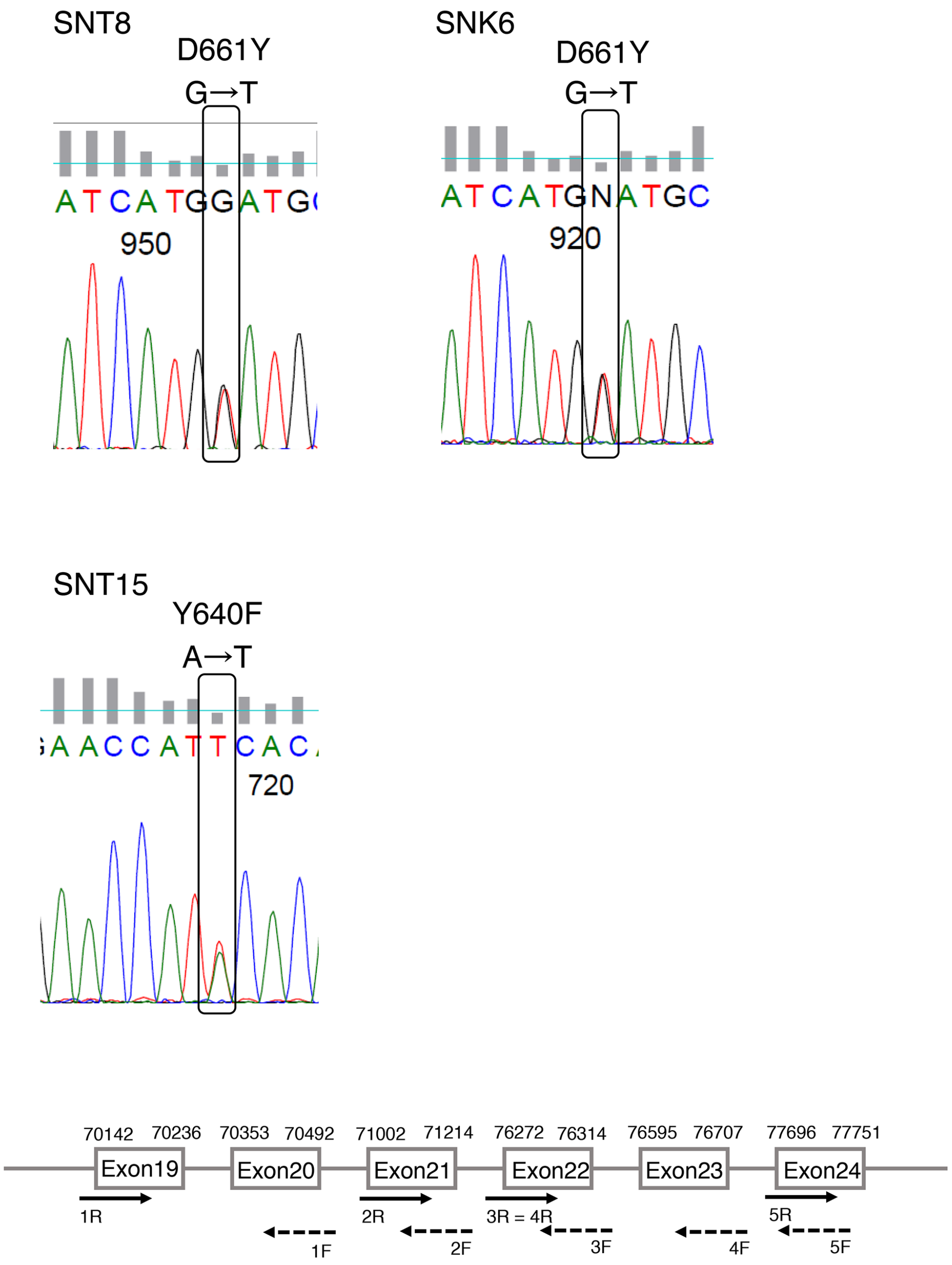

Supplementary Figure 1: Sequencing of the *STAT3* Src homology 2 (SH2) domain of 6 cell lines and 12 patients with CAEBV. SNT8 and SNK6 demonstrated D661Y mutation and SNT15 exhibited Y640F mutation. CAEBV cases did not reveal any *STAT3* SH2 domain mutations.

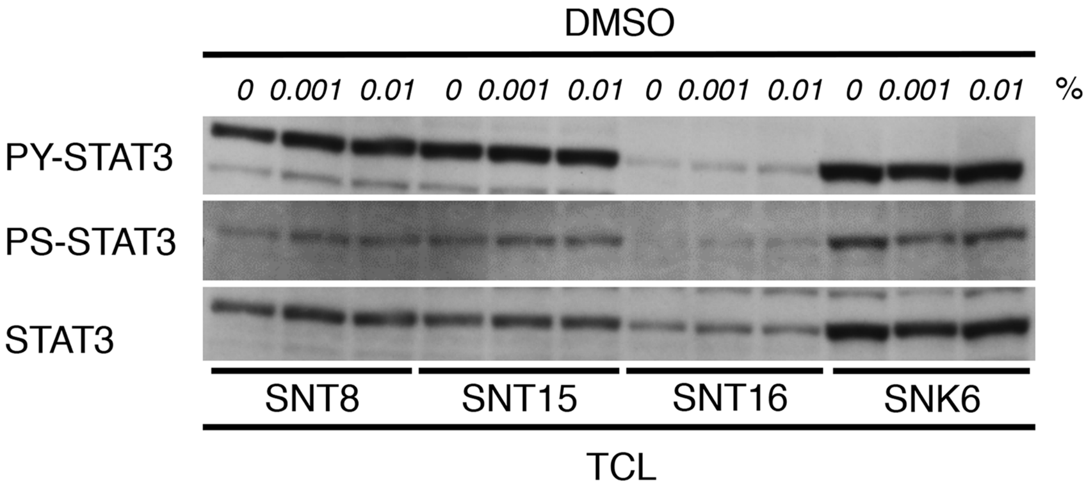

Supplementary Figure 2: Four cell lines were treated with DMSO at the identical concentration of the inhibitors. The phosphorylation of tyrosine-705 and serine-727 were not downregulated by the treatment.

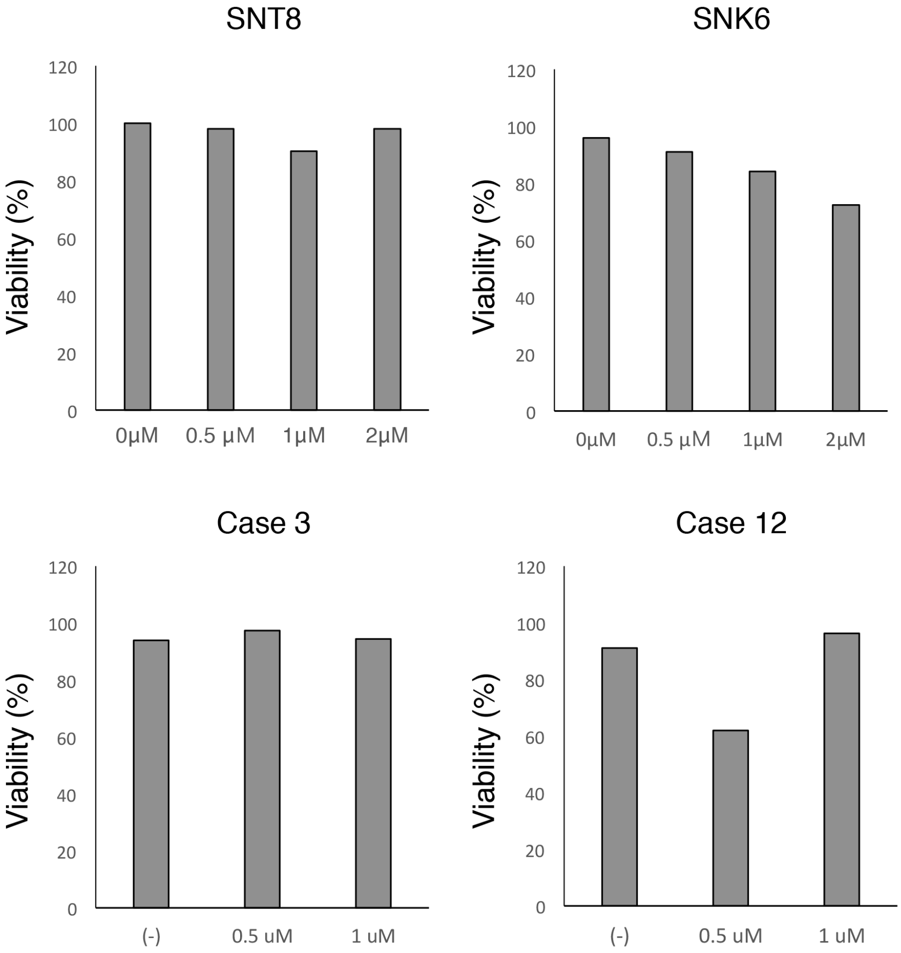

Supplementary Figure 3: The viability for the cells of Figure 5.
